# Near-infrared spectrometry in pregnancy: progress and perspectives, a review of literature

**DOI:** 10.11604/pamj.2016.23.39.5857

**Published:** 2016-02-12

**Authors:** Anouar Jarraya, Smaoui Mohamed, Laabidi Sofiene, Kamel Kolsi

**Affiliations:** 1 Hedi Chaker University Hospital, Sfax, Tunisia

**Keywords:** Near-Infrared Spectrometry, pregnancy, preeclamsia, cesarean section

## Abstract

Near-infrared spectroscopy (NIRS) allows continuous noninvasive monitoring of in vivo oxygenation in selected tissues. It has been used primarily as a research tool for several years, but it is seeing wider application in the clinical arena all over the world. It was recently used to monitor brain circulation in cardiac surgery, carotid endarteriectomy, neurosurgery and robotic surgery. According to the few studies used NIRS in pregnancy, it may be helpful to assess the impact of severe forms of preeclampsia on brain circulation, to evaluate the efficacy of different treatments. It may also be used during cesarean section to detect earlier sudden complications. The evaluation of placental function via abdominal maternal approach to detect fetal growth restriction is a new field of application of NIRS.

## Introduction

Near-infrared spectroscopy (NIRS) is a relatively old tool [1] that allows continuous noninvasive monitoring of in vivo oxygenation in selected tissues such as muscle and brain [1, 2]. NIRS can determine changes in tissue oxygenation based on variable absorption of near-infrared (NIR) light by hemoglobin, myoglobin, and cytochrome c oxidase [2]. So, it allows the evaluation of oxygen availability and oxygen consumption relationships within a living tissue [3]. It has been used primarily as a research tool for several years, but it is seeing wider application in the clinical arena all over the world [4, 5] as a comprehensive monitor of regional oxygen metabolism [6, 7]. In obstetrics, NIRS is not commonly used in spite of several specific complications of pregnancy that may suddenly affect maternal brain circulation such as hypertensive encephalopathy [8], intracerebral hemorrhage [8], cardiopulmonary shock caused by amniotic fluid embolism [9], massive blood loss [10] in adition to compilations from the anesthesia such as hypotension or desaturation [11]. However, recent studies [12–15] showed NIRS as a promising and useful monitoring of tissue oxygenation in pregnant women. The aim of this review is to explain the progress in using NIRS in pregnant women monitoring and its perspectives.

## Methods

A literature search was conducted with the assistance of a senior researcher. The archives of the National Library of Medicine (PubMed) including the Ovid Medline databases were searched. The search was limited to the English and french languages. The articles were selected by reviewing their titles and abstracts with additional references identified from the reference lists of selected articles.

## Current status of knowledge

### Near-Infrared Spectrometry (NIRS) and clinical applications

NIRS is an old tool dating back to 1977 used initially as noninvasive neuroimaging tool that can measure local hemodynamic changes in the brain [1]. It has been used primarily as a research tool for several years [1], but over the past decade, the technical advantage offered by NIRS has provided various new findings on higher cerebral function of the human brain. These findings have improved our knowledge of cognitive neuroscience [16], neurology [17, 18], psychiatric medicine [19–21], rehabilitation medicine [21], and pharmacology [22]. Nowadays, NIRS is widely used as a monitoring in surgical patients. It is used during cardiopulmonary bypass to monitor cerebral circulation [23]. It was also used to monitor the effect of hypotension induced by anesthesia induction [24] and the effects of different vasopressors used to correct this hypotension on brain circulation [24, 25]. Vascular surgery needing arterial clamping providing ischemic lesions is the best indication of NIRS that allows a monitoring of tissue oxygenation in order to avoid ischemia [26–28].

In Robotic assisted prostatic surgery an extreme Trendelenburg position and CO2 pneumoperitoneum are necessary, which may lead to cerebral oedema witch can potentially reduce brain perfusion and therefore could impair cerebral oxygenation. The NIRS seems to be very useful in this case [29]. In obstetrics, the use of NIRS is still limited and there are only few studies [12–15] showing some benefits. This is what we will detail in the rest of the article.

### NIRS in preeclampsia

Although the patho physiology is not fully understood, preeclampsia is a multisystem disorder that may result in substantial maternal complications, such as acute renal failure, pulmonary edema, coagulation impairment or hemolysis, HELLP syndrome [30], and neonatal problems and deaths [31]. Neurologic symptoms are often associated with severe forms of preeclampsia and may ultimately progress to eclampsia [30, 32]. Although cerebrovascular complications are uncommon occurrences during pregnancy and the puerperium, stroke is still the most common seriously disabling complication of pregnancy. Therefore, stroke and other vascular issues (eclampsia) raise questions about the best evaluation and management that is safe for mother and child [12, 32]. At present, cerebral function can be assessed by clinical symptoms and brain imaging. transcranial Doppler (TCD) or ultrasonography have been used to assess cerebral injuries in SP or eclampsia but are not routinely performed [33–35].

Cerebral oximetry seems to be safe for mother and child and seems to be helpful to assess maternal brain circulation but it has never been used to investigate the neurological status of severe preeclamptic parturients before the recent study of Philippe Guerci [12]. Guerci and al [12] were the first who showed that cerebral oxygenation impairment in severe preeclamsia parturients can be detected by near-infrared spectroscopy monitoring. They showed that neurological signs observed in preeclampsia may alter cerebral microcirculation with subsequent decreased cerebral oxygenation. They also found that MgSO4 infusion in patients with severe preeclampsia restored rcSo2 to control levels.

Preeclampsia can be complicated by posterior reversible encephalopathy syndrome (PRES) [36, 37] which is the result of a vasogenic edema caused by cerebral autoregulation breakthrough with subsequent hyperperfusion, blood brain barrier disruption, and endothelial cell dysfunction [32, 38]. The contribution of frontal NIRS may be limited. Further studies are needed to confirm the utility of near-infrared spectroscopy monitoring in patients with posterior reversible encephalopathy and to assess the action of antihypertensive therapies (Labetolol, nicardipine …etc) on rcSo2.

### NIRS in cesarean section

NIRS was recently used to study the effect of spinal anesthesia with hyperbaric bupivacaine or isobaric bupivacaine for elective cesarean section on cerebral blood oxygenation changes [13]. Kondo et al [13] found that isobaric bupivacaine may be superior to hyperbaric bupivacaine for preventing a decrease of maternal cerebral blood flow after spinal anesthesia for cesarean section. Fassoulaki et al [39] showed that rSO2 left and right frontal area values decreased significantly from baseline with most remarkable decreases 5 and 10 minutes after spinal injection. However, the clinical impact of these results remains to be determined. By contrast, TRS-20, a new near-infrared time-resolved spectroscopic system, has high data acquisition and can calculate tissue oxygen saturation by evaluating the absolute concentrations of oxygenated, deoxygenated and total haemoglobin through measuring the transit time of photons through a tissue of interest [40–42]. Kaori et al assessed cerebral oxygen saturation by TRS-20 in pregnant women during cesarean section and showed that it is a promising new method of maternal monitoring [15].

They also showed that In massive bleeding, Cerebral oxygen saturation decreased soon while it was not associated with the changes of fluid loading, administration of phenylephrine, nor procedures of anaesthesia and operation. Nevertheless, these critical changes did not affect maternal peripheral oxygen saturation measured by pulse oximetry, frequently used by anaesthesiologists [15]. In the same study[15], there was also four cases of pre-eclampsia witch have chronic changes with elevated base levels of cerebral oxygen saturation, though peripheral oxygen saturation was similar to that in normotensive pregnant women. So, TRS-20 could detect acute as well as chronic changes in brain oxygen saturation in response to pregnancy-associated complications [15]. Until today there is no study used NIRS to improve the survival of amniotic fluid embolism although it seems useful [43].

### NIRS and the evaluation of placental function

Placental morphological abnormalities and some umbilical cord abnormalities may be prenatally detected using ultrasonography [44]. However, it is still difficult to evaluate placental function and its relationship to fetal growth restriction [44]. Only a few studies have applied NIRS for placental function via the maternal abdominal approach [14, 45, 46]. In a recent study [14], Hasegawa et al included 282 delivered neonates of appropriate for gestational age and 44 small for gestational age babies. The measurement of the concentration of oxyhemoglobin and deoxyhemoglobin was conducted using transabdominal NIRS targeting the placenta at around 20 weeks, 30 weeks and after 36 weeks of gestation and calculated tissue oxygen indexes. This study showed that tissue oxygen indexes measured immediately before delivery in the “small for gestational age” group with severe pre-eclampsia (79.2+/-3.8; P=0.002) and placental abnormalities (78.2+/-3.6; P=0.043) were higher than in the “appropriate for gestational age” group (74.0+/-4.5)and it was lower in case of in the umbilical cord abnormalities in “small for gestational age” group(69.7+/-7.7; P=0.024) in comparison with the “appropriate for gestational age” group.

## Conclusion

NIRS is seeing increased clinical application. It seems to be useful in pregnancy. It was used to assess the impact of severe forms of preeclampsia on brain circulation, to evaluate the efficacy of different treatments and to detect earlier its neurological complications [12]. It may also be used during cesarean section to assess the impact of anesthesia induction [13] or operative incidents such as important bleeding [15]. It was also useful in evaluation of placental function via abdominal maternal approach to detect fetal growth restriction [14]. The NIRS is recently used in pregnancy and other studies may widen the fields of application in pregnancy.

### What is known about this topic

- NIRS allowed continuous noninvasive monitoring of oxygenation in the brain.

### What this study adds

- NIRS can be used to assess the impact of preeclampsia on brain circulation.
- NIRS can be used to evaluate the efficacy of different treatments of preeclampsia.
- NIRS can be used to evaluate the placental function.
